# Varieties of the Pulse

**Published:** 1871-10

**Authors:** Charles W. Chancellor

**Affiliations:** Professor of Anatomy in the Washington University (Medical School), Baltimore, Md.


### Miscellaneous.

### Varieties of the Pulse.

By CHARLES W. CHANCELLOR, M. D., Professor of Anatomy in the Washington University (Medical School), Baltimore, Md.

The phenomenon called the pulse is inseparable from life in the warm-blooded animals, except in the hybernating during their state of torpor; it exists also in the more perfect of the cold-blooded animals.

In viewing man we observe, independently of any particular anatomical knowledge, a symmetry of form, a gracefulness of motion, and an expression of life and intelligence admirably calculated to attract our attention; and if we were not liable to various accidents and diseases, which cause us to suffer and to die, there would be little inducement to enter further into the study of the human organism. What was probably undertaken, therefore, from feelings of necessity, growing out of the frailties of our structure, has now become so interesting that no organ or function can be examined without exciting admiration, and a desire to know more of the phenomena of animal life.

The researches of two thousand years have served only to excite our curiosity; and though much has been done, we are probably but at the threshold of physiology. We must not. however, forget that through a long course of dark ages nothing was added to physiology, and as nothing like science could be introduced into this department of philosophy until the discovery of the circulation of the blood, it is evident that this science but dawned with the discovery of Harvey at the beginning of the seventeenth century.

We are now perfectly familiar with what is properly termed the circulation—we know that it is only necessary for the venous blood to enter the right auricle of the heart to commence its suit of actions, which shall succeed each other in the healthy state with unerring regularity. The auricle contracting, throws the blood into the corresponding ventricle; it is then forced into the lungs through the pulmonary arteries, returned by the veins to the left auricle and ventricle into the aorta, and thence distributed to the capillaries.

Although it is obvious that there must be a regular correspondence of action between the auricles and ventricles, and although there is an association and concentration of actions in a state of health, still we may readily perceive that in addition to the disturbance which may arise in the heart by a want of proportion between the sensorial and the stimulus of the blood, there may be diseased action from want of proportion as to time, or force, or sensibility, in the moving apparatus of the different cavities. The power of the ventricles may not exactly correspond with that of the auricles, or that of one ventricle with the other ventricle, etc.

After all, so far as we can discover variations in the pulse, they depend in a great measure upon the more or less regularity or perfection with which the left ventricle performs its office.

There has been considerable diversity of opinion among medical writers respecting the pulse, both as to the best arrangement obtained by an examination of the subject. Some have held the opinion that we cannot discriminate with sufficient certainty beween one pulse and another, except as to frequency and force. Of this opinion the celebrated John Hunter, in his Treatise on the Blood, page 218, makes this remark: That "frequence of pulsation in a given time is measurable by instruments; smartness or quickness in the stroke, with a pause, is measurable by the touch; but the nicer peculiarities in the pulse are only sensations in the mind." He continues: "I have been certain of the pulse having a disagreeable jar in it when others did not receive it; when they were only sensible of its frequency and strength; and it is perhaps this jar that is the specific distinction between constitutional disease or irritation and health. Frequency of pulsation may often arise from stimulus, but the stroke will then be soft, yet softness is not to be depended on as a mark of health. It is often a sign of dissolution, but then there must be other attending symptoms."

What Mr. Hunter calls a "jar," Dr. Rush terms a "jerk." No writer has obtained more universal credit for veracity than Dr. Rush, and he says, speaking of the peculiarity of the pulse in the fever of 1793, that he thought he could have distinguished the disease by it without seeing the patient. "This pulse," he says, "attended cases of remittents produced that year by the same causes as yellow fever, and often occurred in persons who were able to go about."

All the well-marked varieties of the pulse may be included in the full, tense, slow, frequent, quick, soft, irregular, and feeble.

*The Full Pulse* is common in phlegmonous inflammations, in inflammations of the pleura, and in acute exanthemata. In these cases, particularly in the athletic, it is also tense, and generally somewhat frequent. In some cases of extreme debility from exhaustion by hæmorrhage, or disease, the pulse is full, mostly frequent, and very yielding and soft. This condition has been termed the compressible pulse.

*The Tense Pulse*, or the hard pulse, we have seen sometimes attend the full pulse, but tension of the arterial coats indicates more danger when the vessel is small and very tense, not because there is a reduced quantity of blood, but because we know by actual observation that this state of the pulse indicates severe disease of some important vital organ. A tense pulse can, under no circumstances, be indicative of anything salutara. It has been remarked by Dr. Rush and others that this pulse is almost always present at the onset of malignant fevers, and this same author tells us that "in some cases the tense and full pulse were present in the last hours of life." A degree of tension of the artery at the wrist, though considerably above the healthy standard, does not particularly indicate danger; but great tension with remarkable slowness, or great tension with extreme frequency, are almost always ominous of danger.

*The Slow Pulse*, when remarkable in acute disease, is perhaps always indicative of danger. It should be remembered that instances of slow pulse occur as idiosyncrasies of habit, or it may be that there are intermediate strokes too feeble to be perceived. In an illness where the pulse suddenly becomes slow from being feverishly quick, with an aggravation of other symptoms, it is an indication of the disease being translated to the brain; or if the pulse of a child be fifteen or twenty below the lowest limit of the natural standard, it may be regarded as a certain indication of the brain being affected. In injuries about the head, too, the slow pulse indicates cerebral complication. A case came under my observation a short time since illustrative of this fact. A lad, twelve years old, recived in a fall a slight blow upon the left parietal boss, from which he experienced no particular inconvenience at the time, but in a few minutes he complained of headache and confused vision. There was no mark of violence upon the scalp, nor of injury to the cranium, but the boy's pulse was only *forty* beats to the minute. This, in itself, was sufficient to awaken a suspicion that "shock" had been communicated to the nervous system, and a course of treatment to meet the indications was adopted. In ten or fifteen minutes thereafter vomiting ensued, the surface became pale and cold, the pupils were widely dilated, and the sufferer passed into a condition of semi-consciousness, with all the objective and subjective indications of concussion. Now, in this instance, the slow pulse was the first, and for some time the only diagnostic sign of cerebral complication, and was in itself sufficient to point out the nature of the injury.

*The Frequent Pulse* does not particularly indicate danger, unless above 120. When upwards, in acute fevers, the patient will generally be delirious, except in hectic. In rheumatism the pulse will frequently beat upwards of 120 without indicating danger, but a pulse upwards of 120 is said to be almost always a fatal omen in asthma. Authors speak of seeing patients recover whose pulse had been 180. This pre-supposes the possibility of counting 180 beats per minute, which can seldom be done; indeed where there is extreme frequency we cannot easily count above 140.

*The Quick Pulse.*--The heart is so easily thrown into irregular action in diseases that nothing is more reasonable than the supposition that it may perform its systolic action in less time than natural, and it is doubtful whether we obtain any advantage from endeavoring to estimate the quickness of the pulse. The difference between the frequent and quick pulse is readily demonstrated, notwithstanding many use these terms as synonymous; the former takes cognizance of the number of beats in a given time, say one minute, the other has relation to the time of contraction to the left ventricle of the heart and the muscular tunic of the artery. Dr. Rush expressly tells us that in the epidemic of 1793 the pulse was both frequent and quick in many instances; and this peculiarity has also been recognized by Good and other authors.

*The Soft Pulse.*—This state of the pulse is, perhaps, never indicative of danger, except in cases of extreme debility; and here the danger is manifested as much by the frequency as by its softness, or rather weakness; when it attends dissolution it is usually under those circumstances. Generally this pulse is the harbinger of returning health; nothing is more acceptable to the experienced physician or surgeon after a state of morbid excitement, from whatever cause, than a soft, equable pulse. In short, it is almost uniformly the pulse of convalescents, but here it is mostly equable.

There is, however, a soft pulse, with very considerable dilatation of the arteries, which sometimes attends a great and sudden degree of exhaustion. This peculiarity is seen where much blood has been lost by hemorrhage. The blood, in these cases, is greatly weakened in its texture; it contains very little plasma or red corpuscles, being principally serum, which will scarcely stain a linen rag.

*The Irregular Pulse* is often seen in malignant fevers and in violent injuries of the viscera, especially the liver; the heart and arteries hobble, as it were, through their functions. This pulse also attends rheumatic diseases, without, however, portending danger; but in malignant fevers or injuries of the viscera it may be regarded as an unfavorable, if not dangerous symptom.

*The Feeble Pulse* is often associated with other states of the pulse. In protracted diseases generally a feeble stroke of the artery will take place. In typhus fever it is a distinguishing symptom, which occurs very early in the disease, and is persistent, whereas in hectic fever it changes with the paroxysms, the pulse being at one time of the day tense, and at another extremely feeble, and it is not till a protracted period that the feebleness predominates.

There are other states of the pulse noticed by different writers, which, however, are without any well-grounded meaning or utility. Sir A. Cooper speaks of an "oppressive state of the pulse"; Dr. Darwin of an "empty pulse"; Dr. Rush of a "shattered or quill pulse." Others have spoken of a "laboring pulse," a "breathing pulse," a "thready pulse," but all these may properly be referred to the foregoing heads.

Lastly, there are instances in which the radial artery in one arm presents abnormities which might lead to error in diagnosis; it will, therefore, be well, at least with strangers, to examine both arms, as other peculiarities may be met with of which patients may be insensible.

Celsus, who was ignorant of the circulation of the blood, characterized the pulse as a *res fallassisima*; but since the brilliant discovery of Harvey its value as a diagnostic means has been universally recognized, and as we become more conversant with the laws or principles by which organs so curious are enabled to preserve their own existence and perform certain functions individually and in unison, the pulse will become more and more a subject of interest and investigation.—*Richmond and Louisville Medical Journal.*

